# Determinants of Child Growth in Palestine (Ages 5–17): A Structural Equation Modeling Approach to Food Insecurity, Nutrition, and Socioeconomic Factors

**DOI:** 10.3390/children12060703

**Published:** 2025-05-29

**Authors:** Suleiman Thwib, Radwan Qasrawi, Ghada Issa, Malak Amro, Razan Abu Ghoush, Sabri Saghir, Doa’a Mujahed, Maysaa Nemer, Mousa Halaika, Manal Badrasawi, Ayoub Al-Jawaldeh, Ibrahim Elmadfa, Lara Nasreddine, Diala Abu Al-Halawa, Maisan Nimer

**Affiliations:** 1 Department of Computer Sciences, Al Quds University, Jerusalem P144, Palestinegissa@staff.alquds.edu (G.I.);; 2 Department of Computer Engineering, Istinye University, 34010 Istanbul, Turkey; 3 The Center of Technology and Innovation, Al-Quds University, Jerusalem P144, Palestine; razan.ghoush@staff.alquds.edu; 4 Department of Nutrition and Food Technology, College of Agriculture, Hebron University, Hebron P720, Palestine; sabrisaghir@hebron.edu (S.S.);; 5 Institute of Community and Public Health, Birzeit University, Ram Allah P606, Palestine; 6 Nutrition Department, Ministry of Health, Ram Allah P606, Palestine; 7 Nutrition and Food Technology Department, Faculty of Agriculture and Vetrenary Medicine, An-Najah National University, Nablus P400, Palestine; m.badrasawi@najah.edu; 8 Regional Office for the Eastern Mediterranean, World Health Organization, Cairo 7608, Egypt; 9 Department of Nutrition, Faculty of Life Sciences, University of Vienna, 1010 Vienna, Austria; 10 Nutrition and Food Sciences Department, Faculty of Agriculture and Food Sciences, American University of Beirut, Beirut 1107 2020, Lebanon; 11 Department of Medicine, Al-Quds University, Jerusalem P144, Palestinemaisan.nimer@students.alquds.edu (M.N.)

**Keywords:** child growth, structural equation modeling, food insecurity, micronutrients, socioeconomic status, Palestine, nutrition

## Abstract

**Background**: The growth patterns of children and adolescents are influenced by multiple factors. This study employed structural equation modeling (SEM) to determine the primary factors influencing the growth of Palestinian children and adolescents in the West Bank (WB). **Methods**: A cross-sectional survey conducted in 2022 in the WB collected data from 1400 households, of which 500 with children aged 5–17 years and were selected for analysis. The survey assessed household food insecurity, socioeconomic status (SES), parental health history, nutritional awareness, food consumption patterns, and nutrient intake. The latent construct “Child Growth” was measured using Body Mass Index-for-age Z-score (BAZ), height-for-age Z-score (HAZ), and Mean Adequacy Ratio (MAR). SEM was employed to examine the interrelations among latent variables and their observed indicators. **Results**: Mineral intake showed the strongest direct effect on child growth (β = 0.812, *p* < 0.001), followed by food availability (β = 0.492), vitamin intake (β = 0.481), SES (β = 0.439), and macronutrient intake (β = 0.383). MAR exhibited the highest factor loading as a growth indicator, while HAZ had limited significance, suggesting its chronic nature. SES influenced growth both directly and indirectly through its effects on food availability and dietary intake pathways. Strong correlations between nutritional knowledge and nutrient classes reflect the interrelationship between behavioral and biological determinants. **Conclusions**: Both structural and immediate nutritional factors influence child growth. In Palestinian youth, mineral intake, food security, and SES have the greatest influence. These findings offer a framework for understanding the growth determinants of Palestinian youth in the WB and support the development of targeted interventions to improve dietary quality and overall nutritional status.

## 1. Introduction

Child growth is a global indicator of physical well-being and a measure of a typical pattern of changes that ought to occur from infancy to adolescence [1,2]. Data evidence shows that inadequate physical growth is typically linked to impaired or delayed cognitive development and is critical to children’s full mental development [3,4]. In Palestine and other regions affected by armed conflict, noncombatant populations like children are most vulnerable to the loss of food, income, accommodation, and resources essential to their healthy growth and welfare [5]. The ongoing economic and political instability in Palestine have engendered significant challenges for the healthy development of children. The interplay of determinants affecting child growth in Palestine is understudied. Consequently, the literature does not fully explain the factors that should inform actionable interventions to reverse or prevent negative trajectories in healthy growth.

Child growth is influenced by various factors, including food insecurity, nutritional deficiencies, and socioeconomic disparities [4,6]. The complexity of these interrelated factors makes structural equation modeling (SEM) a valuable analytical tool for identifying their effects on children’s growth, as it allows for the simultaneous assessment of direct and indirect relationships between multiple variables. For instance, research conducted by Jebena et al. in Ethiopia demonstrated a significant association between food insecurity and poor mental health, growth trajectories, and overall human capital outcomes [7]. By employing SEM, the study showed that household food insecurity mediates the relationship between socioeconomic status and child development. These findings align with other studies that identified food insecurity as a significant predictor of common mental disorders among Ethiopian youth, further emphasizing its impact on child well-being [8,9,10]. The role of dietary diversity in mitigating the effects of food insecurity was highlighted by several studies, where their analysis showed that children with higher dietary diversity scores exhibited better academic performance and nutritional outcomes, regardless of socioeconomic status [11,12,13].

Nutritional factors, including dietary quality, and their relationship with socioeconomic status (SES) and child growth have been widely studied, with consistent findings indicating that economic disparities significantly impact health outcomes. A study by Huang et al. utilized SEM to investigate childhood obesity in relation to family dietary habits and socioeconomic factors. The findings indicated that parental education and economic stability were positively associated with healthier food consumption patterns and lower childhood obesity rates [14]. Similar results were reported by Chea et al., who found that food insecurity and low SES were strongly linked to poor dietary habits and increased consumption of unhealthy foods among children [15]. In Malaysia, Cheah et al. explored the relationship between socioeconomic determinants and child malnutrition using SEM, revealing that maternal education and access to healthcare were key factors influencing nutritional status, with higher maternal education levels correlating with improved child health outcomes [15]. Dwomoh et al. further expanded on this by examining how dietary practices and household environmental quality mediated socioeconomic inequalities in child undernutrition risk in West Africa [16]. Their SEM model demonstrated that improving dietary diversity and environmental hygiene could significantly reduce child malnutrition rates. Hartwell et al. also applied SEM to assess the impact of income, parental education, and food security on child development in the United States [17]. Their results indicated that parental education had the strongest positive influence on growth indicators, while food insecurity had an indirect negative effect through reduced dietary diversity. In a similar vein, Riddle et al. examined the associations between empowerment dimensions and nutritional status among married adolescent girls in East Africa, finding that higher levels of education and autonomy were positively linked to improved nutritional outcomes [18].

Traditionally, indicators such as Body Mass Index (BMI) and Mean Nutritional Adequacy Ratio (MAR) have been employed to assess the nutritional status and growth outcomes in children and adolescents from the ages of 5 to 17 [19]. BMI is a screening tool for macro nutritional status, such as undernutrition, normal range, or overnutrition. It offers a standardized measure that facilitates comparisons across different populations and over time. Some limitations include that it does not measure body fat distribution, and BMI alone may not capture the subtleties of nutritional deficiencies or excesses, especially in situations where undernutrition and overnutrition coexist. The Mean Nutritional Adequacy Ratio is a metric that expresses an individual’s average nutrient consumption as a percentage of the corresponding recommended amount. As a result, MAR is more effective for identifying micronutrient deficiencies that are not always apparent through BMI measurements. Combining these indicators with structural equation modeling provides a powerful framework for examining the complex relationships between socioeconomic status, food security, and child growth [20,21,22].

Despite extensive research on child growth determinants, little is known about how socioeconomic factors, household food insecurity, nutritional knowledge, dietary practices, and nutrient intake interact to influence child development in Palestine. The country faces unique challenges, including economic difficulties and political instability, which worsen food insecurity and limit access to essential nutrition. This study utilized structural equation modeling (SEM) to analyze how the aforementioned factors collectively influence child growth outcomes in Palestinian children aged 5–17 years.

## 2. Materials and Methods

### 2.1. Data Source

This study utilized primary data from a cross-sectional survey conducted in the West Bank, Palestine, in 2022 [23]. The survey was specifically designed to evaluate household food insecurity, socioeconomic conditions, nutritional knowledge, dietary behaviors, and parental practices related to child nutrition and growth. Initially, data were collected from 1400 households, but only 500 included children and adolescents aged between 5 and 17 years. For this specific analysis, only data from these 500 households were utilized, focusing on the detailed examination of growth determinants using structural equation modeling.

To address missing data in the dataset, we used full information maximum likelihood (FIML) estimation. FIML allows all available data to be used in the analysis without the need to delete cases with missing values, which helps preserve sample size and re-duce bias. This approach is appropriate under the assumption that data are missing at random (MAR), which we considered reasonable after reviewing the patterns and extent of missingness in our variables. FIML is a commonly used method in structural equation modeling and has been shown to provide reliable estimates even with moderate levels of missing data.

### 2.2. Study Variables

Data were gathered using structured interviews. The assessed variables are presented in Table 1.

### 2.3. Data Analyses

Descriptive statistics for continuous variables were summarized using means and standard deviations, while categorical variables were reported as frequencies and percentages. Univariate correlation analyses were conducted to explore associations among growth indicators and nutritional outcomes, including height for age, BMI for age, and Mean Inadequate Nutrient Intake Ratio, based on WHO growth standards for children aged 5–19 years [24].

Prior to model fitting, the distributional properties of observed variables were assessed using skewness and kurtosis statistics. The results of this normality assessment informed the selection of the appropriate estimation method for subsequent analyses.

Confirmatory factor analysis (CFA) was performed to validate the measurement model [26,27]. CFA was used to assess the construct validity and reliability of latent variables by examining factor loadings, average variance extracted (AVE), and composite reliability (CR) [28]. Criteria for acceptable validity in CFA included standardized factor loadings greater than 0.500 (with significance at *p* < 0.05), AVE values exceeding 0.500, and an AVE for each latent variable that was greater than its squared correlation with any other latent variable. CR values exceeding 0.6 indicated acceptable reliability [26,28].

Subsequently, structural equation modeling (SEM) was applied to identify the determinants influencing child growth outcomes by analyzing direct and indirect relationships among socioeconomic factors, nutritional knowledge, dietary behaviors, nutrient intake, and household food insecurity in Palestinian children aged 5–17 years in the West Bank. SEM analyses were conducted using AMOS 20 software [29]. Model fit was assessed using chi-square (χ^2^), normed fit index (NFI), comparative fit index (CFI), and root mean square error of approximation (RMSEA). A good model fit was indicated by a non-significant χ^2^ test, NFI and CFI values of at least 0.950, and RMSEA values of 0.070 or lower. Furthermore, the maximum likelihood (ML) estimation method was employed when variables were normally or approximately normally distributed [26,27,28]. In cases of slight deviations from normality, ML remained appropriate, whereas Bayesian estimation was utilized when substantial deviations were present.

## 3. Results

This section presents findings on determinants of child growth among Palestinian children aged 5–17 years using structural equation modeling (SEM). Results are divided into descriptive analysis, highlighting anthropometric measures (BAZ, HAZ) and nutritional adequacy (MAR) across sociodemographic and dietary factors, and SEM analysis, which examines interrelationships between socioeconomic status, food availability, nutrient intake, parental health, nutritional awareness, and their impacts on child growth outcomes.

### 3.1. Descriptive Analysis

Results in Table 2 shows the distribution of growth indicators across socioeconomic factors. Regarding locality, refugee camps showed the highest proportion of children below the recommended standards for both BAZ (24.5%) and HAZ (17%), showing a higher level of nutritional vulnerability compared to cities and villages. Villages followed closely, particularly in BAZ (23.8%), indicating persistent nutritional challenges.

#### 3.1.1. Anthropometric vs. Socioeconomic Data

Moderate-income families exhibited the highest percentage of substandard BAZ (24.5%), while low-income families had a substantial but lower proportion (21.3%). Moderate-income households also had a notably high percentage of children below the HAZ standard (15.4%), significantly higher than that of high-income families (5.6%), suggesting that even moderate financial resources may not fully protect children from nutritional deficiencies.

The employment status of parents revealed minimal differences. Nonetheless, children with employed fathers showed slightly higher proportions of substandard BAZ (25.8%) compared to those with unemployed fathers (22.9%). Regarding maternal employment, children of unemployed mothers experienced marginally higher rates of substandard nutrition (23% BAZ, 13.6% HAZ below standard) than those of employed mothers.

Gender-based differences showed that males were slightly more at risk of being below BAZ standards (25.6%) than females (21.5%), while HAZ deficiencies were slightly more common among males (13.9%) compared to females (11.3%). Mothers’ education levels showed that secondary education correlated with slightly higher proportions of below-standard BAZ (24.1%) compared to primary education (21.8%), but HAZ outcomes were comparable, indicating a limited influence of maternal education levels on child nutrition within this context.

Age group analysis demonstrates a clear pattern: adolescents aged 13–17 were most vulnerable to being below BAZ standards (27.2%), whereas younger children aged 5–8 exhibited lower rates (18.9%). However, HAZ deficiency was most prominent in younger age groups (5–8, 13.4%; 9–12, 12.8%).

Family size had noticeable implications: medium-sized families (5–6 members) had higher proportions of children below the BAZ standards (24.7%) compared to smaller families (2–4 members, 20.2%). HAZ deficiencies were most prevalent in larger families with seven or more members (16.9%), suggesting that limited resources may adversely affect long-term nutritional status.

#### 3.1.2. Macronutrient Intake Adequacy

The nutrient intake data were compared to the U.S. National Research Council’s Recommended Dietary Allowances, which set nutrient requirements for children based on their age, gender, and body measurements to determine whether their diets are sufficient, excessive, or inadequate [30].

Table 3 shows macronutrient intake adequacy among children, compared by age and gender. Regarding energy intake, inadequacy was notably higher among children aged 5–8 years (41.7%), while it decreased with age (21.8% for 9–12 years and 20.4% for 13–17 years). Energy inadequacy was slightly higher among males (30.6%) compared to females (24.5%). In contrast, protein inadequacy increased with age: children aged 13–17 years had the highest inadequacy (30.2%), followed by those aged 9–12 years (19.9%) and 5–8 years (6.3%). Females exhibited slightly greater inadequacy (21.1%) compared to males (17.8%). Carbohydrate inadequacy, while lower overall, was slightly more common among children aged 5–8 years (21.3%) than among older groups (14.7% for 9–12 years and 12.3%, respectively for 13–17 years). Gender-based inadequacy was nearly identical between males (15.6%) and females (15.8%).

Fat intake inadequacy was a significant concern, affecting nearly half of all children. Children aged 5–8 years had the highest proportion of inadequate fat consumption (54.3%), followed closely by those aged 9–12 years (53.8%), whereas adolescents aged 13–17 years showed a lower but still notable rate (39.5%). Females (49.4%) and males (47.8%) showed similar inadequacy levels, indicating that low fat consumption is widespread across genders. Fiber inadequacy was relatively high across all age groups, consistently exceeding 90%, with only marginal differences observed among age groups (ranging from 92% to 92.3%). However, males displayed slightly higher inadequacy (94.4%) compared to females (90.6%).

#### 3.1.3. Vitamin Intake Adequacy

Vitamin intake was compared to the RDAs, which define age, gender, and size specific needs to assess if diets meet recommended levels [30].

Table 4 shows vitamin intake adequacy among children, compared by age and gender. Folate inadequacy was particularly severe among younger children aged 5–8 years, affecting 89.8% of this age group, while remarkably dropping to 0% among children aged 9–12 years and remaining very low (1.9%) in adolescents aged 13–17. Gender disparities were minor, with males slightly more affected (28.3%) than females (24.9%). Vitamin A inadequacy followed a similar pattern, dramatically affecting younger children aged 5–8 years (95.3%) and declined to negligible levels in the older age groups. Again, males experienced slightly higher inadequacy (30%) compared to females (26.4%).

Vitamin B-complex inadequacies varied significantly across individual vitamins and age groups. Vitamin B1 inadequacy was consistently high, ranging from 66% among children aged 9–12 years to 74.1% among adolescents aged 13–17 years, indicating widespread and persistent inadequacy across all ages and genders. Vitamin B2 inadequacy increased substantially with age, rising from 25.2% in younger children to 66.7% in adolescents aged 13–17, reflecting growing nutritional gaps in older age groups. Females showed marginally higher inadequacy (49.1%) than males (46.7%). Furthermore, inadequate intake of vitamin B3 (niacin) was high across all age groups, progressively increasing with age from 61.4% among the youngest to 80.9% in adolescents. In terms of gender, females displayed slightly higher inadequacy (74%) compared to males (70%). Vitamin B5 (pantothenic acid) inadequacy similarly showed a significant increase with age, from 29.1% among younger children to 79% among adolescents aged 13–17. This trend signals a growing risk of deficiency in older children. Males and females had similar inadequacy levels (61.1% and 60.4%, respectively).

Vitamin B6 inadequacy showed similar patterns observed in folate and vitamin A, severely impacting younger children (96.1%) but virtually nonexistent among older groups. Males (30%) were slightly more vulnerable compared to females (26.8%). Vitamin B12 inadequacy was consistently high across all age groups, averaging around 71%. The inadequacy was similar across both genders, with females (71.3%) and males (70.6%) equally affected. Vitamin C inadequacy increased with age, with adolescents (81.5%) most affected, followed closely by children aged 9–12 years (75%) and those aged 5–8 years (73.2%). Gender differences were minimal, with males (76.7%) slightly less affected than females (77%).

#### 3.1.4. Mineral Intake Adequacy

Mineral intake was evaluated against RDAs to determine dietary adequacy [30]. Table 5 shows the mineral and nutrient intake adequacy among children, compared by age and gender. Inadequacies were found particularly in calcium, magnesium, potassium, and phosphorus. Calcium inadequacy was extremely high across all age groups, starting from 92.1% among younger children (5–8 years) and nearly universal among adolescents (99.4%). This inadequacy showed minimal gender differences. Magnesium inadequacy increased markedly with age, from 55.1% in younger children to almost universal inadequacy (98.8%) among adolescents. Both genders faced similar inadequacy levels, highlighting magnesium intake as a significant nutritional concern as children grow older. Potassium inadequacy was consistently severe, affecting nearly all children (99.3%), with negligible differences by age or gender, reflecting a critical and common dietary gap. Phosphorus inadequacy similarly increased with age, from 37.8% in younger children to 94.4% among adolescents. Although females had slightly higher inadequacy than males, the overall issue was severe, particularly in older children.

Manganese inadequacy presented a unique pattern; younger age groups had low inadequacy rates (around 7%), sharply rising to 91.4% among adolescents. Gender differences were minimal, indicating that age rather than gender drives manganese inadequacy. Iron inadequacy was significant, particularly high among adolescents (72.8%) and younger children (65.4%), while slightly better among 9–12-year-olds. Females experienced marginally higher inadequacy compared to males, pointing to persistent iron intake challenges, especially during adolescence. Zinc inadequacy steadily worsened with age, rising from 46.5% among the youngest group to 75.9% in adolescents. Males and females experienced similar inadequacy levels, emphasizing the growing nutritional deficit with age.

### 3.2. SEM Analysis

#### 3.2.1. Model Fit

To examine the relationships between socioeconomic factors, food availability, eating patterns, macronutrient intake, mineral and vitamin consumption, parental health, nutritional awareness, and child growth outcomes, a structural equation model was developed and tested. The overall model fit was assessed using multiple goodness-of-fit indices as presented in Table 6.

The ratio of chi-square to degrees of freedom (χ^2^/df = 1.924) indicated good fit, as it is well below the recommended threshold of 3.0. The root mean square error of approximation (RMSEA = 0.046, 90% CI [0.043, 0.048]) indicated good model fit, with the narrow confidence interval indicating precision in the estimate. The PCLOSE value of 0.998 provided strong evidence that the model represents a close fit to the data.

The standardized root mean square residual (SRMR = 0.015) demonstrated an excellent fit, indicating minimal differences between observed and model-implied correlations.

Although the comparative fit index (CFI = 0.827) and Tucker–Lewis Index (TLI = 0.817) fall slightly below the conventional threshold of 0.90, these values are still considered acceptable in the context of complex models involving latent constructs and multiple pathways, particularly in public health and social research. Given the theoretical coherence of the model and acceptable fit across other indices (e.g., RMSEA, SRMR), we retained the current structure while acknowledging this limitation and suggesting it as an area for future refinement. Furthermore, the goodness-of-fit index (GFI = 0.809) indicated an acceptable fit, while the adjusted goodness-of-fit index (AGFI = 0.792) was marginally acceptable, approaching the 0.80 threshold.

#### 3.2.2. Normality Test

**Figure 1:**
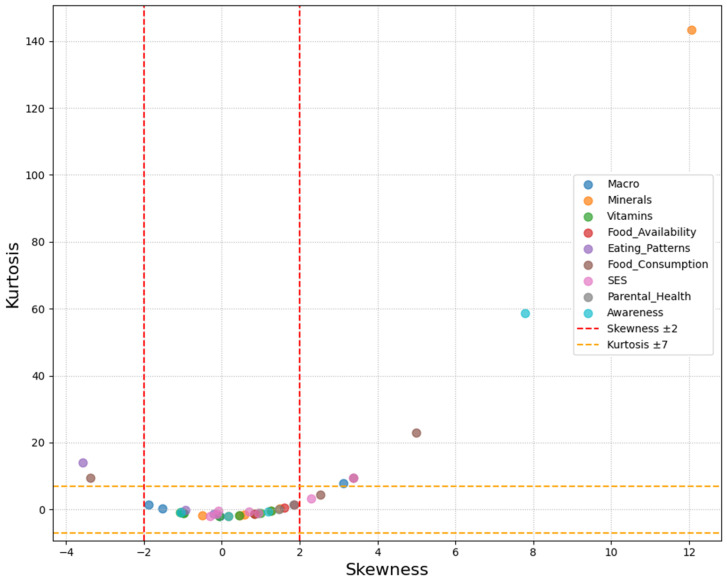
Distribution of skewness and kurtosis for observed variables, colored by construct.

The assessment of data normality revealed that most variables demonstrated acceptable distributional properties, though several showed notable departures from normality. Among all the examined variables, extreme positive skewness was observed in potassium intake (skew = 12.056), knowledge of sweet problems (skew = 7.784), and food aid dependency (skew = 3.855), indicating that most participants had low values, with few having high levels. Conversely, negative skewness was evident in macronutrient variables, particularly carbohydrate (skew = −1.883) and protein intake (skew = −1.518), and meal timing patterns such as lunch consumption (skew = −3.568). These distributional characteristics are visually summarized in Figure 1.

Kurtosis analysis revealed heavy-tailed distributions for several variables, with potassium showing the most extreme values (kurtosis = 143.340), followed by calcium intake (kurtosis = 29.261) and knowledge variables. The multivariate normality test confirmed a significant departure from normality (1012.506, c.r. = 132.503), which was expected given the categorical nature of the variables.

Despite these departures from normality, the distributional characteristics were deemed acceptable for structural equation modeling analysis using robust maximum likelihood estimation, which is specifically designed to handle non-normal data commonly encountered in behavioral and nutritional research.

#### 3.2.3. SEM Diagram

**Figure 2:**
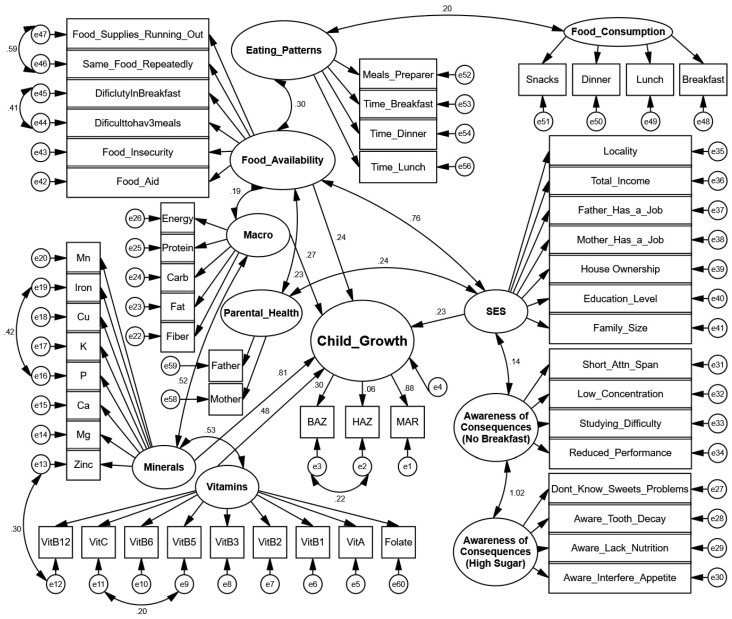
Structural equation model of determinants of child growth in Palestinian children aged 5–17 years.

The structural equation model path diagram depicts the complex relationships between multiple latent constructs affecting child growth outcomes. As illustrated in Figure 2, child growth is conceptualized as a latent variable measured through three observed indicators: Body Mass Index-for-age Z-score (BAZ), height-for-age Z-score (HAZ), and Mean Adequacy Ratio (MAR). These indicators represent different aspects of growth and nutritional status, with BAZ reflecting weight relative to height, HAZ measuring linear growth, and MAR assessing overall nutritional adequacy.

The path diagram highlights several key structural relationships. Socioeconomic status (SES) serves as a fundamental upstream determinant, measured through multiple indicators including locality, total income, parental employment status, house ownership, education level, and family size. SES shows a substantial direct effect on food availability (path coefficient = 0.76), indicating that higher socioeconomic status significantly improves household food security. SES also directly influences child growth (path coefficient = 0.23), indicating socioeconomic factors contribute independently to growth outcomes beyond other pathways.

Food availability, measured through indicators of food insecurity, food aid dependence, and dietary challenges, demonstrates important relationships with eating patterns (path coefficient = 0.30) and macronutrient intake (path coefficient = 0.19). The model also captures two domains of nutritional awareness: awareness of the consequences of skipping breakfast and awareness of the consequences of high sugar consumption, with the latter showing a strong relationship with the former (path coefficient = 1.02).

Nutritional pathway analysis shows that minerals have a substantial direct effect on child growth (path coefficient = 0.81), significantly stronger than the effect of vitamins (path coefficient = 0.22). Minerals are represented by multiple micronutrients, including manganese, iron, copper, potassium, phosphorus, calcium, magnesium, and zinc, while vitamins include several B vitamins, vitamins A and C, and folate. The relationship between vitamins and minerals shows a considerable correlation (0.53), indicating their interrelated role in nutrition. Regarding child growth indicators, the model shows that BAZ and MAR have stronger loadings (0.81 and 0.88, respectively) compared to HAZ, which has a notably weaker loading (0.06). To validate the inclusion of HAZ in the growth construct, nested model comparisons were conducted between models with and without HAZ. The removal of HAZ did not significantly affect the overall model fit indices (ΔCFI < 0.01, ΔRMSEA < 0.005) or other parameter estimates, confirming its minimal contribution to the latent growth variable. However, HAZ was retained to maintain theoretical completeness of the anthropometric assessment and to capture important correlational relationships, which may reflect underlying causal pathways between acute and chronic malnutrition indicators.

#### 3.2.4. Latent-Indicator Standardized Direct Effects

The results in Table 7 present the standardized direct effects between the various latent constructs and their respective indicator variables in the structural equation model, along with their statistical significance. The analysis of direct effects on child growth indicated that minerals had the strongest influence (β = 0.812, *p* < 0.001), followed by food availability (β = 0.492, *p* = 0.029), vitamins (β = 0.481, *p* < 0.001), SES (β = 0.439, *p* = 0.038), and macronutrients (β = 0.383, *p* < 0.001). Among the child growth indicators, MAR showed the strongest loading (β = 0.883, *p* < 0.001), followed by BAZ (β = 0.295, *p* < 0.001). The non-significant loading of HAZ (β = 0.065, *p* = 0.216) indicates that HAZ had little direct effect on children’s growth.

To better understand the weak role of height-for-age Z-score (HAZ) in the growth construct, we ran an additional test comparing two versions of our model: one that included HAZ and another that left it out. The results showed only a very small and statistically non-significant difference between the two models, meaning that keeping or removing HAZ did not meaningfully change the overall fit of the model.

For socioeconomic status (SES), total income emerged as the strongest indicator (β = 0.666, *p* < 0.001), followed by father’s employment (β = 0.463, *p* < 0.001), locality (β = 0.388, *p* < 0.001), mother’s employment (β = 0.278, *p* < 0.001), and family size (β = 0.145, *p* = 0.018). Within the food availability construct, food insecurity was the strongest indicator (β = 0.773, *p* < 0.001), followed by food aid dependency (β = 0.328, *p* < 0.001), breakfast difficulty (β = 0.272, *p* < 0.001), and repetitive food consumption (β = 0.175, *p* = 0.003).

Among macronutrients, protein showed the strongest loading (β = 0.785, *p* < 0.001), followed by carbohydrates (β = 0.539, *p* < 0.001), fat (β = 0.445, *p* < 0.001), and fiber (β = 0.261, *p* < 0.001). For vitamins, vitamin B6 (β = 1.001, *p* < 0.001), vitamin A (β = 0.994, *p* < 0.001), folate (β = 0.955, *p* < 0.001), vitamin B5 (β = 0.408, *p* < 0.001), vitamin B2 (β = 0.29, *p* < 0.001), and vitamin B3 (β = 0.15, *p* = 0.001) emerged as significant indicators. The mineral construct was significantly represented by phosphorus (β = 0.744, *p* < 0.001), magnesium (β = 0.731, *p* < 0.001), zinc (β = 0.607, *p* < 0.001), manganese (β = 0.49, *p* = 0.009), iron (β = 0.449, *p* < 0.001), copper (β = 0.341, *p* < 0.001), calcium (β = 0.26, *p* = 0.001), and potassium (β = 0.149, *p* < 0.001). In the parental health domain, the father’s disease status was a significant indicator (β = 0.644, *p* < 0.001).

#### 3.2.5. Latent-Indicator Standardized Indirect Effects

The results in Table 8 present the standardized indirect effects between key latent constructs and the child growth indicators (BAZ, HAZ, and MAR) in the structural equation model, based on 200 bootstrap samples with bias-corrected 95% confidence intervals. The analysis of indirect effects reveals how various determinants influence specific growth indicators through mediating pathways. For Body Mass Index-for-Age Z-score (BAZ), minerals showed the strongest indirect effect (β = 0.24), followed by macronutrients (β = 0.179), SES (β = 0.135), vitamins (β = 0.075), and food availability (β = 0.071). These indirect effects show that minerals and macronutrients play important mediating roles in the pathways leading to weight-related growth outcomes.

For HAZ, all indirect effects were notably smaller compared to other growth indicators, with minerals having the largest indirect effect (β = 0.064), followed by vitamins (β = 0.036), food availability (β = 0.022), and macronutrients (β = 0.017). The Mean Adequacy Ratio (MAR) showed the strongest indirect effects across all variables, with minerals having a particularly substantial indirect effect (β = 0.947), followed by vitamins (β = 0.526), food availability (β = 0.322), and macronutrients (β = 0.251). These strong indirect effects on the MAR highlight the cumulative impact of various nutritional pathways on overall nutritional adequacy.

#### 3.2.6. Inter-Construct Covariances

The analysis of covariances in Table 9 shows important correlational relationships between key constructs. The strongest relationship was observed between awareness of breakfast consequences and awareness of high sugar consequences (r = 1.02, *p* = 0.002), indicating that these two aspects of nutritional knowledge are highly interrelated.

Socioeconomic status (SES) showed a strong correlation with food availability (r = 0.76, *p* < 0.001), highlighting the substantial relationship between household economic resources and food security. SES also correlated significantly, though more modestly, with parental health (r = 0.23, *p* = 0.027) and awareness of breakfast consequences (r = 0.14, *p* = 0.041). Food availability showed significant correlations with parental health (r = 0.23, *p* = 0.014) and macronutrient intake (r = 0.19, *p* < 0.001), as well as a marginally significant relationship with eating patterns (r = 0.30, *p* = 0.072).

Strong nutritional correlations were observed between vitamins and minerals (r = 0.53, *p* < 0.001) and between macronutrients and minerals (r = 0.52, *p* < 0.001). Additional nutritional correlations were observed between vitamin B12 and zinc (r = 0.30, *p* < 0.001), iron and phosphorus (r = 0.42, *p* < 0.001), and vitamin B5 and vitamin C (r = 0.20, *p* < 0.001). Regression analyses confirmed these nutrient domains remain distinct predictors of MAR with acceptable multicollinearity, as indicated by variance inflation factors (VIFs 1.1–4.2), supporting their treatment as separate but interrelated constructs in the model.

Food insecurity indicators were also significantly correlated, with a strong relationship between food supplies running out and consuming the same food repeatedly (r = 0.59, *p* < 0.001), as well as between breakfast difficulty and not having three meals daily (r = 0.41, *p* < 0.001). Notably, while the direct effect of the model’s variables on HAZ was weak, the significant correlation between BAZ and HAZ (r = 0.22, *p* < 0.001) indicates an important relationship between these two anthropometric measures.

## 4. Discussion

This study wanted to assess the relationships between socioeconomic factors, household food insecurity, nutritional knowledge, dietary practices, and nutrient intake, as well as their collective impact on child growth outcomes of Palestinian children in the West Bank. Significant nutritional disparities were found among children influenced by location, socioeconomic status, and age. Children in refugee camps exhibited the highest prevalence of substandard BAZ and HAZ, likely due to limited access to diverse, nutrient-rich foods and healthcare services. Displacement-related constraints are well documented and proven to worsen food insecurity and undernutrition [31,32].

Contrary to expectations, children from moderate-income families showed higher BAZ and HAZ deficiencies than those from low-income households, who probably rely mainly on available fortified wheat flour products. This unexpected finding may be due to a shift in food consumption patterns where increased income leads to consuming more low-nutrient, calorie-dense foods instead of a higher-quality diet [33]. Moreover, moderate-income families may fall outside the scope of food aid programs, increasing their nutritional risk.

Parental employment and maternal education had minimal influence, suggesting that financial resources and education alone may not be sufficient without corresponding improvements in food accessibility and health literacy [34]. Age played a critical role: older children exhibited more acute undernutrition (low BAZ), whereas chronic growth failure (low HAZ) was more common among younger children, reflecting their distinct developmental needs. Macronutrient data support these trends. Younger children showed higher energy and fat inadequacies, possibly due to insufficient intake or poor food variety, while adolescents faced greater protein shortfalls, reflecting increased physiological needs during puberty [35]. Fat and fiber inadequacies were widespread, indicating a lack of dietary diversity and low intake of whole foods. Micronutrient analysis revealed severe deficiencies, particularly among younger children for folate, vitamin A, and B6, which are critical for early development. In contrast, B-complex and vitamin C inadequacies increased with age, suggesting a dietary transition that excludes nutrient-dense foods as children grow older [36]. Persistent B12 and iron deficiencies, especially among adolescents and females, raise concerns for cognitive and physical development [37]. Mineral inadequacies, including calcium, magnesium, and potassium, were nearly common, with severity increasing by age. These results emphasize cumulative dietary inadequacy, likely due to reliance on energy-dense but micronutrient-poor diets [36].

The structural equation model (SEM) demonstrated an acceptable fit for explaining the complex pathways influencing child growth, particularly in contexts where multiple socioeconomic and nutritional variables interact. Although some fit indices were marginally below ideal thresholds, such values are acceptable in complex behavioral models with latent constructs [38]. Child growth was best represented by indicators reflecting recent nutritional status, namely dietary adequacy and weight for age. Height for age contributed weakly to the model, which is consistent with its role as a chronic indicator of long-term undernutrition and early-life deprivation; this supports evidence that stunting, unlike acute malnutrition, is less responsive to short-term dietary or socioeconomic changes [39,40]. Nutritional adequacy emerged as a stronger construct than physical growth measures alone. This aligns with findings that nutrient intake, particularly the adequacy of vitamins and minerals, is critical for metabolic function, immune competence, and cognitive development [6,41]. Macronutrient intake, while relevant, plays a more supportive role, as growth relies heavily on the bioavailability and interaction of micronutrients.

The model shows that socioeconomic status (SES) is a key determinant of child growth, exerting both direct and indirect effects through food availability and nutrient intake. Total income and paternal employment proved to be the most influential SES indicators. Economic constraints shape dietary diversity and household food security, both of which are key mediators in the nutritional pathway [34]. These findings are supported by life-course models of child development, which argue that parental income and occupation determine not only food purchasing power but also access to health services, environmental sanitation, and early childhood development inputs [34]. The strong association between SES and food availability illustrates how structural inequality influences food security. Lower-SES groups often face constraints in both food quantity and quality, with higher dependency on food aid or repetitive, low-nutrient diets. The findings align with global data from the FAO (2022), where household food insecurity correlates strongly with socioeconomic deprivation, particularly in displacement and low-resource settings [42]. Food availability, in turn, demonstrated meaningful effects on macronutrient intake and eating patterns, reinforcing its mediating role between SES and child growth. Households reporting food shortages or meal monotony were more likely to have children with inadequate dietary intake. These findings can be explained by the concept of “hidden hunger”, where caloric needs may be met, but micronutrient intake remains insufficient or even deficient due to a lack of dietary diversity [35]. Furthermore, food insecurity, captured strongly by the indicator “food running out”, directly compromises not only nutrient intake but also feeding frequency and quality.

Among all pathways, micronutrient intake, particularly minerals, was the most powerful predictor of child growth. This result is strongly justified by the essential roles that minerals such as phosphorus, zinc, and magnesium play in biological processes, including bone mineralization, cellular metabolism, immune function, and enzymatic activity. For example, zinc deficiency impairs DNA replication and protein synthesis, directly hindering tissue growth and repair [35]. Similarly, phosphorus is vital for skeletal development and energy metabolism [43]. The high indirect effect of minerals on MAR further emphasizes the cumulative contribution of minerals adequacy to overall dietary sufficiency.

Vitamins also contributed significantly to growth, though to a lesser extent compared to minerals. Vitamin B6, vitamin A, and folate showed the highest loadings within this domain. This finding is consistent with the role of B vitamins in metabolic regulation, erythropoiesis, and nervous system function, as well as with the known association of vitamin A deficiency with impaired epithelial integrity and immune competence [44]. The observed intercorrelation between vitamins and minerals reflects the biological synergy between these nutrient classes in supporting cellular processes. The role of macronutrients was also evident, with protein as the dominant contributor. This aligns with protein’s role in tissue synthesis, hormone production, and immune modulation. The lower path coefficients for fat and fiber are still relevant, as fat is the source of essential fatty acids, facilitates fat-soluble vitamin absorption, and provides dense energy for growing children, while fiber supports gut health and nutrient absorption [45].

The employed SEM further showed differential effects on growth indicators: MAR and BAZ were more responsive to nutritional pathways than HAZ. This distinction supports the theory that acute malnutrition (reflected in BAZ and MAR) is more influenced by current dietary intake, whereas HAZ responds to long-term exposures, including infection, inflammation, and intergenerational malnutrition [45,46]. The strong covariance between awareness of breakfast and sugar consequences indicates that nutrition knowledge domains cluster tightly, possibly influenced by shared educational or public health messaging. Yet, their limited direct effect on child growth implies that knowledge must be coupled with actionable resources and behavior change strategies to influence outcomes [47]. Moreover, the significant correlation between BAZ and HAZ, despite HAZ’s weak direct effect, indicates partial overlap in the biological and environmental factors shaping both linear and weight-for-height growth. This further reinforces the multifactorial nature of child undernutrition, where acute and chronic growth disturbances coexist and interact.

## 5. Conclusions

This study identifies inadequate mineral intake as the strongest direct predictor of child growth (β = 0.812, *p* < 0.001), followed by food availability (β = 0.492, *p* = 0.029), vitamin intake (β = 0.481, *p* < 0.001), socioeconomic status (SES) (β = 0.439, *p* = 0.038), and macronutrient intake (β = 0.383, *p* < 0.001). Minerals such as phosphorus (β = 0.744), magnesium (β = 0.731), and zinc (β = 0.607) were particularly influential, underscoring the role of micronutrient sufficiency in supporting metabolic and skeletal development. Food insecurity mediated SES effects by limiting dietary diversity and meal frequency, while SES itself exerted both direct and indirect effects on growth via improved food access and nutrient quality. Together, these findings highlight that interventions targeting micronutrient fortification and food security enhancement in low-resource, conflict-affected settings like the West Bank are critical to ameliorating child malnutrition.

This study offers a comprehensive analysis of the multidimensional determinants of child growth using a structural equation modeling (SEM) approach. The model demonstrated a good overall fit and effectively captured the interrelationships between socioeconomic status, food availability, nutrient intake, and child nutritional outcomes. Socioeconomic status emerged as a foundational determinant, exerting both direct and indirect effects on food security and growth. However, nutritional factors, particularly mineral intake, were identified as the most influential direct predictors of child growth, notably in shaping overall nutritional adequacy and weight-related outcomes. The weak association between height for age and immediate dietary variables reinforces its role as a chronic indicator of long-term nutritional deprivation, less responsive to short-term dietary improvements. The findings also underscore the synergistic effects among micronutrients, macronutrients, and household-level determinants, highlighting the need for multisectoral strategies. Effective interventions should prioritize improving minerals and vitamin intake through dietary diversification, food fortification, and supplementation, while simultaneously addressing structural socioeconomic barriers to food access. Our model outlines critical drivers of child malnutrition, offering actionable insights for nutrition policies and programs in resource-limited settings. Future work should apply the same approach to examine the determinants of infant and toddler growth under the age of five, as well as the interplay between physical and cognitive development and how each informs or affects the other.

## 6. Study Limitations

This study offers a valuable contribution to understanding the multifactorial drivers of child malnutrition in conflict-affected settings. However, the cross-sectional design of this study limits our ability to draw causal inferences, as the structural equation modeling reflects only contemporaneous associations rather than changes over time. Additionally, since data were drawn exclusively from 500 households in the West Bank, our findings may not generalize to other regions of Palestine (such as Gaza) or to different cultural and conflict-affected settings. Furthermore, dietary intake was assessed via two 24 h recalls (one weekday and one weekend), which are vulnerable to recall bias and may not accurately represent habitual or seasonal variations in nutrient consumption. Moreover, key variables, such as household food insecurity, nutritional knowledge, and certain demographic characteristics, were self-reported and therefore susceptible to social desirability and reporting biases. Finally, while overall model fit indices were acceptable (RMSEA = 0.046; SRMR = 0.015), the comparative fit index (CFI = 0.827) and Tucker–Lewis index (TLI = 0.817) fell below conventional thresholds (≥0.90), suggesting that additional latent variables or alternate model specifications might improve explanatory power.

## Figures and Tables

**Table 1:** Study variables.

Construct	Variables
Child Growth	Children’s growth estimated based on the WHO growth reference data from 5 to 19 years. The growth construct is composed of BMI for age; height for age; Mean Nutrient Inadequate Ratio [24].
Socioeconomic and Demographic	Age, gender, household income, family size, parental education, employment status, and refugee status.
Household Food Insecurity	Radimer/Cornell Hunger Scale (food quantity, food quality, food acceptability, certainty of obtaining food) [25].
Dietary Intake	Two 24 h dietary recalls (weekday and weekend), types and amounts of food/beverages consumed, meal timings, locations, preparation methods, macronutrients and micronutrients (e.g., magnesium, potassium, phosphorus, iron, zinc), and comparison with RDA standards.
Anthropometric Measures	Height, weight, waist circumference, Body Mass Index (BMI) based on WHO and International Obesity Task Force criteria.
Nutrition-related Practices	Dietary habits, meal patterns, food purchasing and preparation practices, autonomy in food-related decisions.
Awareness of Skipping Breakfast Consequences	Recognition of attention span impact, school performance, and general knowledge of breakfast importance.
Knowledge of High Sugar Intake Risks	Awareness of tooth decay, nutritional deficiency, and appetite interference.
Attitude Toward Meal Regularity	Importance assigned to breakfast and regular meals and snacks.
Practical Barriers to Eating Regularly	Difficulties experienced in having breakfast regularly and maintaining consistent meal patterns.
Actual Meal Consumption	Consumption patterns of breakfast, lunch, dinner, and snacks.
Meal Timing and Regularity	Timing consistency for breakfast, lunch, and dinner.
Meal Preparation and Consumption Environment	Locations of meal consumption and person responsible for meal preparation.
Autonomy in Food Purchasing	Independent food purchasing behaviors and purchasing locations.
Food Shortage and Insufficiency	Food shortage experience and economic barriers affecting portion size and meal frequency.

**Table 2:** Distribution of anthropometric status (BAZ and HAZ) by socioeconomic and demographic factors among Palestinian children aged 5–17 Years.

		BMI–Age Z-Score			Height–Age Z-Score		
		Below	Normal	Above	Below	Normal	Above
Locality	City	51 (22.4)	155 (68)	22 (9.6)	24 (10.5)	194 (85.1)	10 (4.4)
	Village	39 (23.8)	114 (69.5)	11 (6.7)	22 (13.4)	132 (80.5)	10 (6.1)
	Refugee Camp	13 (24.5)	36 (67.9)	4 (7.5)	9 (17)	42 (79.2)	2 (3.8)
Family Income	Low	10 (21.3)	34 (72.3)	3 (6.4)	6 (12.8)	40 (85.1)	1 (2.1)
	Moderate	67 (24.5)	182 (66.7)	24 (8.8)	42 (15.4)	218 (79.9)	13 (4.8)
	High	26 (20.8)	89 (71.2)	10 (8)	7 (5.6)	110 (88)	8 (6.4)
Father Job	No	95 (22.9)	284 (68.6)	35 (8.5)	51 (12.3)	343 (82.9)	20 (4.8)
	Yes	8 (25.8)	21 (67.7)	2 (6.5)	4 (12.9)	25 (80.6)	2 (6.5)
Mother Job	No	73 (23)	217 (68.5)	27 (8.5)	43 (13.6)	258 (81.4)	16 (5)
	Yes	30 (23.4)	88 (68.8)	10 (7.8)	12 (9.4)	110 (85.9)	6 (4.7)
gender	Male	46 (25.6)	120 (66.7)	14 (7.8)	25 (13.9)	146 (81.1)	9 (5)
	Female	57 (21.5)	185 (69.8)	23 (8.7)	30 (11.3)	222 (83.8)	13 (4.9)
Mother education	Primary	41 (21.8)	126 (67)	21 (11.2)	22 (11.7)	154 (81.9)	12 (6.4)
	Secondary	62 (24.1)	179 (69.6)	16 (6.2)	33 (12.8)	214 (83.3)	10 (3.9)
Age	5–8	24 (18.9)	95 (74.8)	8 (6.3)	17 (13.4)	104 (81.9)	6 (4.7)
	9–12	35 (22.4)	101 (64.7)	20 (12.8)	20 (12.8)	127 (81.4)	9 (5.8)
	13–17	44 (27.2)	109 (67.3)	9 (5.6)	18 (11.1)	137 (84.6)	7 (4.3)
Family Members	2–4	22 (20.2)	81 (74.3)	6 (5.5)	8 (7.3)	95 (87.2)	6 (5.5)
	5–6	48 (24.7)	130 (67)	16 (8.2)	23 (11.9)	165 (85.1)	6 (3.1)
	7+	33 (23.2)	94 (66.2)	15 (10.6)	24 (16.9)	108 (76.1)	10 (7)

**Table 3:** Distribution of macronutrient intake adequacy among children compared by age and gender.

		5–8 Years	9–12 Years	13–17 Years	Male	Female	Total
Energy	Inadequate	53 (41.7)	34 (21.8)	33 (20.4)	55 (30.6)	65 (24.5)	120 (27)
	Adequate	74 (58.3)	122 (78.2)	129 (79.6)	125 (69.4)	200 (75.5)	325 (73)
Protein	Inadequate	8 (6.3)	31 (19.9)	49 (30.2)	32 (17.8)	56 (21.1)	88 (19.8)
	Adequate	119 (93.7)	125 (80.1)	113 (69.8)	148 (82.2)	209 (78.9)	357 (80.2)
Carb	Inadequate	27 (21.3)	23 (14.7)	20 (12.3)	28 (15.6)	42 (15.8)	70 (15.7)
	Adequate	100 (78.7)	133 (85.3)	142 (87.7)	152 (84.4)	223 (84.2)	375 (84.3)
Fat	Inadequate	69 (54.3)	84 (53.8)	64 (39.5)	86 (47.8)	131 (49.4)	217 (48.8)
	Adequate	58 (45.7)	72 (46.2)	98 (60.5)	94 (52.2)	134 (50.6)	228 (51.2)
Fiber	Inadequate	117 (92.1)	144 (92.3)	149 (92)	170 (94.4)	240 (90.6)	410 (92.1)
	Adequate	10 (7.9)	12 (7.7)	13 (8)	10 (5.6)	25 (9.4)	35 (7.9)

**Table 4:** Distribution of vitamin and nutrient intake adequacy among children compared by age and gender.

		5–8 Years	9–12 Years	13–17 Years	Male	Female	Total
Folate	Inadequate	114 (89.8)	0 (0)	3 (1.9)	51 (28.3)	66 (24.9)	117 (26.3)
	Adequate	13 (10.2)	156 (100)	159 (98.1)	129 (71.7)	199 (75.1)	328 (73.7)
VitA	Inadequate	121 (95.3)	0 (0)	3 (1.9)	54 (30)	70 (26.4)	124 (27.9)
	Adequate	6 (4.7)	156 (100)	159 (98.1)	126 (70)	195 (73.6)	321 (72.1)
VitB1	Inadequate	85 (66.9)	103 (66)	120 (74.1)	124 (68.9)	184 (69.4)	308 (69.2)
	Adequate	42 (33.1)	53 (34)	42 (25.9)	56 (31.1)	81 (30.6)	137 (30.8)
VitB2	Inadequate	32 (25.2)	74 (47.4)	108 (66.7)	84 (46.7)	130 (49.1)	214 (48.1)
	Adequate	95 (74.8)	82 (52.6)	54 (33.3)	96 (53.3)	135 (50.9)	231 (51.9)
VitB3	Inadequate	78 (61.4)	113 (72.4)	131 (80.9)	126 (70)	196 (74)	322 (72.4)
	Adequate	49 (38.6)	43 (27.6)	31 (19.1)	54 (30)	69 (26)	123 (27.6)
VitB5	Inadequate	37 (29.1)	105 (67.3)	128 (79)	110 (61.1)	160 (60.4)	270 (60.7)
	Adequate	90 (70.9)	51 (32.7)	34 (21)	70 (38.9)	105 (39.6)	175 (39.3)
VitB6	Inadequate	122 (96.1)	0 (0)	3 (1.9)	54 (30)	71 (26.8)	125 (28.1)
	Adequate	5 (3.9)	156 (100)	159 (98.1)	126 (70)	194 (73.2)	320 (71.9)
VitB12	Inadequate	90 (70.9)	110 (70.5)	116 (71.6)	127 (70.6)	189 (71.3)	316 (71)
	Adequate	37 (29.1)	46 (29.5)	46 (28.4)	53 (29.4)	76 (28.7)	129 (29)
VitC	Inadequate	93 (73.2)	117 (75)	132 (81.5)	138 (76.7)	204 (77)	342 (76.9)
	Adequate	34 (26.8)	39 (25)	30 (18.5)	42 (23.3)	61 (23)	103 (23.1)

**Table 5:** Distribution of minerals nutrient intake adequacy among children compared by age and gender.

		5–8 Years	9–12 Years	13–17 Years	Male	Female	Total
Calcium	Inadequate	117 (92.1)	154 (98.7)	161 (99.4)	172 (95.6)	260 (98.1)	432 (97.1)
	Adequate	10 (7.9)	2 (1.3)	1 (0.6)	8 (4.4)	5 (1.9)	13 (2.9)
Mg	Inadequate	70 (55.1)	125 (80.1)	160 (98.8)	142 (78.9)	213 (80.4)	355 (79.8)
	Adequate	57 (44.9)	31 (19.9)	2 (1.2)	38 (21.1)	52 (19.6)	90 (20.2)
Mn	Inadequate	9 (7.1)	12 (7.7)	148 (91.4)	72 (40)	97 (36.6)	169 (38)
	Adequate	118 (92.9)	144 (92.3)	14 (8.6)	108 (60)	168 (63.4)	276 (62)
P	Inadequate	48 (37.8)	140 (89.7)	153 (94.4)	134 (74.4)	207 (78.1)	341 (76.6)
	Adequate	79 (62.2)	16 (10.3)	9 (5.6)	46 (25.6)	58 (21.9)	104 (23.4)
K	Inadequate	126 (99.2)	154 (98.7)	162 (100)	179 (99.4)	263 (99.2)	442 (99.3)
	Adequate	1 (0.8)	2 (1.3)	0 (0)	1 (0.6)	2 (0.8)	3 (0.7)
Cu	Inadequate	76 (59.8)	79 (50.6)	87 (53.7)	103 (57.2)	139 (52.5)	242 (54.4)
	Adequate	51 (40.2)	77 (49.4)	75 (46.3)	77 (42.8)	126 (47.5)	203 (45.6)
Iron	Inadequate	83 (65.4)	72 (46.2)	118 (72.8)	109 (60.6)	164 (61.9)	273 (61.3)
	Adequate	44 (34.6)	84 (53.8)	44 (27.2)	71 (39.4)	101 (38.1)	172 (38.7)
Zinc	Inadequate	59 (46.5)	103 (66)	123 (75.9)	114 (63.3)	171 (64.5)	285 (64)
	Adequate	68 (53.5)	53 (34)	39 (24.1)	66 (36.7)	94 (35.5)	160 (36)

**Table 6:** Goodness-of-fit indices for the structural equation model (SEM) of the determinants of child growth among Palestinian children aged 5–17 years.

Fit Index	Value	Interpretation
χ^2^/df	(2912.34/1514) 1.924	Good fit: <3.0 indicates good fit
CFI	0.827	Acceptable fit: ≥0.80 is acceptable, ≥0.90 is good
TLI	0.817	Acceptable fit: ≥0.80 is acceptable, ≥0.90 is good
RMSEA	0.046	Good fit: <0.05 good, <0.08 reasonable
RMSEA 90% CI	[0.043, 0.048]	Good fit: upper bound <0.08 indicates good precision
PCLOSE	0.998	Excellent: >0.05 suggests close fit
SRMR/RMR	0.015	Excellent fit: <0.05 excellent, <0.08 good
GFI	0.809	Acceptable fit: ≥0.80 is acceptable, ≥0.90 is good
AGFI	0.792	Marginally acceptable: close to 0.80 threshold

**Table 7:** Standardized direct effects and statistical significance in the structural equation model of child growth determinants.

Causal Variable	Effect Variable <--	Direct Effect	Significance (*p*-Value)
Child Growth	SES	0.439	0.038
	Food availability	0.492	0.029
	Macronutrients	0.383	<0.001
	Vitamins	0.481	<0.001
	Minerals	0.812	<0.001
	BAZ	0.295	<0.001
	HAZ	0.065	0.216
	MAR	0.883	<0.001
SES	Locality	0.388	<0.001
	Total income	0.666	<0.001
	Father has a job	0.463	<0.001
	Mother has a job	0.278	<0.001
	Family size	0.145	0.018
	House ownership	0.058	0.322
	Education level	0.033	0.567
Food Availability	Insecurity level	0.773	<0.001
	Breakfast difficulty	0.272	<0.001
	Not having 3 meals	0.093	0.697
	Food aid	0.328	<0.001
	Same food repeatedly	0.175	0.003
	Supplies running out	0.104	0.074
Macronutrients	Protein	0.785	<0.001
	Carb	0.539	<0.001
	Fat	0.445	<0.001
	Fiber	0.261	<0.001
Vitamins	Vitamin A	0.994	<0.001
	Vitamin B1	0.061	0.195
	Vitamin B2	0.29	<0.001
	Vitamin B3	0.15	0.001
	Vitamin B5	0.408	<0.001
	Vitamin B6	1.001	<0.001
	Vitamin B12	0.01	0.845
	Folate	0.955	<0.001
Minerals	Calcium	0.26	0.001
	Magnesium	0.731	<0.001
	Manganese	0.49	0.009
	Phosphorus	0.744	<0.001
	Potassium	0.149	<0.001
	Copper	0.341	<0.001
	Iron	0.449	<0.001
	Zinc	0.607	<0.001
Parental Health	Father has a disease	0.644	<0.001
	Mother has a disease	0.381	0.169
Awareness (Breakfast)	Short attention span	0.556	<0.001
	Low concentration	0.349	<0.001
	Studying difficulty	0.51	<0.001
	Reduced performance	0.552	<0.001
Awareness (High Sugar)	Aware of tooth decay	0.338	0.003
	Aware it lacks nutrition	0.544	0.001
	Aware it interferes with appetite	0.195	0.016

**Table 8:** Standardized indirect effects on child growth indicators.

Causal Variable	Effect Variable <--	S.E. (95% BC CI)	Indirect Effect
BAZ	SES	0.109	0.135
	Food Availability	0.103	0.071
	Macronutrients	0.044	0.179
	Vitamins	0.036	0.075
	Minerals	0.050	0.24
HAZ	Food Availability	0.036	0.022
	Macronutrients	0.024	0.017
	Vitamins	0.026	0.036
	Minerals	0.044	0.064
MAR	Food Availability	0.017	0.322
	Macronutrients	0.112	0.251
	Vitamins	0.080	0.526
	Minerals	0.055	0.947

**Table 9:** Significant standardized covariances in the structural equation model.

Causal Variable	Effect Variable <-->	Total Effect	S.E.	Significance (*p*-Value)
SES	Food Availability	0.76	0.01	<0.001
	Awareness (Breakfast)	0.14	0.004	0.041
	Parental Health	0.23	0.007	0.027
Food Availability	Eating Patterns	0.30	0.007	0.072
	Parental Health	0.23	0.006	0.014
	Macronutrients	0.19	0.006	<0.001
Food Consumption	Eating Patterns	0.20	0.003	<0.001
BAZ	HAZ	0.22	0.027	<0.001
Macronutrients	Minerals	0.52	0.004	<0.001
Vitamins	Minerals	0.53	0.008	<0.001
Vitamin B5	Vitamin C	0.20	0.009	<0.001
Vitamin B12	Zinc	0.30	0.009	<0.001
Iron	Phosphorus	0.42	0.007	<0.001
Breakfast Difficulty	Not Having 3 Meals	0.41	0.011	<0.001
Same Food Repeatedly	Supplies Running Out	0.59	0.008	<0.001
Awareness (Breakfast)	Awareness (High Sugar)	1.02	0.002	0.002

## Data Availability

The data supporting the findings of this study are available from the corresponding authors upon reasonable request. Due to ongoing analyses, the dataset is not publicly accessible at this stage.
